# Cardiovascular evaluation in overweight and obese dogs

**DOI:** 10.29374/2527-2179.bjvm004426

**Published:** 2026-06-19

**Authors:** Giovanna Pestana Silva, Diana do Amaral Mendonça, Emmanuel José Fialho Camilo, Nathália da Conceição Lima, Markos Panayotis Damatis, Rafaela Pereira de Souza, Bruno Alberigi

**Affiliations:** 1 Instituto de Veterinária (IV), Universidade Federal Rural do Rio de Janeiro (UFRRJ), Seropédica, RJ, Brazil; 2 Programa de Pós-Graduação em Medicina Veterinária, IV, UFRRJ. Seropédica, RJ, Brazil.; 3 Programa de Residência em Medicina Veterinária – Clínica Médica de Animais de Companhia. DMCV, IV, UFRRJ. Seropédica, RJ, Brazil.; 4 Programa de Residência em Medicina Veterinária – Cardiologia e Doenças respiratórias. DMCV, IV, UFRRJ. Seropédica, RJ, Brazil.; 5 Departamento de Medicina e Cirurgia Veterinária (DMCV),IV, UFRRJ. Campus Seropédica, RJ, Brazil.

**Keywords:** body condition score, body mass index, echocardiography, electrocardiography, obesity, escore de condição corporal, índice de massa corporal, ecocardiograma, eletrocardiograma, obesidade

## Abstract

Canine obesity is a multifactorial nutritional disorder characterized by excessive accumulation of adipose tissue with systemic health consequences. However, the cardiovascular effects of obesity in dogs have not been completely characterized. This study aimed to evaluate the clinical, electrocardiographic, and echocardiographic parameters of dogs with normal body condition, overweight, and obesity. Thirty client-owned dogs were divided into three groups of ten: Group 1 (G1, body condition score [BCS] 4–5/9), Group 2 (G2, overweight, BCS 6–7/9), and Group 3 (G3, obese, BCS 8–9/9). All animals underwent physical examination, electrocardiography, and echocardiography, and their canine body mass index and thoracic-to-abdominal circumference ratio were measured. Exercise intolerance and dyspnea were significantly more frequent in G3 (90% and 70%) than in G1 (20% and 10%) and G2 (60% and 10%) (p<0.05). The P-wave (p = 0.030) and QRS (p = 0.049) durations increased progressively from G1 to G3. The left atrial diameter (p = 0.010) and left atrium-to-aortic root ratio (p = 0.011) were significantly greater for G3, and mitral valve abnormalities were more prevalent in obese dogs (p = 0.023). The thoracic-to-abdominal circumference ratio was significantly lower in G2 and G3 (p = 0.003), reflecting abdominal fat redistribution. No significant differences were found in the systolic or conventional diastolic function indices. These findings indicate that obesity in dogs is associated with early cardiovascular alterations, morphometric changes, electrocardiographic conduction delays, and left atrial remodeling in the absence of overt cardiac dysfunction. This underscores the importance of routine cardiovascular monitoring in overweight and obese canine patients.

## Resumo

A obesidade canina é uma desordem nutricional multifatorial caracterizada pelo acúmulo excessivo de tecido adiposo com consequências sistêmicas. Os efeitos cardiovasculares da obesidade em cães permanecem, no entanto, incompletamente caracterizados. Este estudo avaliou parâmetros cardiovasculares clínicos, eletrocardiográficos e ecocardiográficos em cães com condição corporal normal, sobrepeso e obesidade. Trinta cães foram divididos em três grupos de dez: Grupo 1 (G1, ECC 4–5/9), Grupo 2 (G2, sobrepeso, ECC 6–7/9) e Grupo 3 (G3, obesos, ECC 8–9/9). Todos foram submetidos a exame físico, à determinação do índice de massa corporal canino (IMCC) e da razão circunferência torácica/abdominal (CT/CA), à eletrocardiografia e à ecocardiografia. Intolerância ao exercício e dispneia foram mais frequentes no G3 (90% e 70%) que no G1 (20% e 10%) e G2 (60% e 10%) (p < 0,05). A duração da onda P (p = 0,030) e do QRS (p = 0,049) aumentaram progressivamente de G1 a G3. O diâmetro atrial esquerdo (p = 0,010) e a relação AE/Ao (p = 0,011) foram significativamente maiores no G3, e alterações da valva mitral foram mais prevalentes nos cães obesos (p = 0,023). A razão CT/CA foi significativamente menor nos grupos G2 e G3 (p = 0,003), o que reflete redistribuição de gordura abdominal. Não foram observadas diferenças significativas nos índices convencionais de função sistólica ou diastólica. Os achados indicam que a obesidade em cães está associada a alterações cardiovasculares precoces, mudanças morfométricas, atrasos de condução eletrocardiográfica e remodelamento atrial esquerdo, na ausência de disfunção cardíaca manifesta, reforçando a importância do monitoramento cardiovascular em pacientes com sobrepeso e obesidade.

## Introduction

Canine obesity is a multifactorial condition that represents one of the main contemporary challenges in maintaining canine health (Endenburg et al., 2018). It is characterized by excessive accumulation of adipose tissue that compromises the health and well-being of animals, including reduced life expectancy (German, 2006; Zoran, 2010). The increasing incidence of obesity in dogs is associated with the evolving role of dogs as family members, which is often accompanied by inappropriate feeding practices and increased sedentary behavior and leads to nutritional and metabolic imbalances (Courcier et al., 2010; Endenburg et al., 2018; Mao et al., 2013). Obesity is estimated to affect >40% of the global canine population (Montoya-Alonso et al., 2017; Thomson et al., 2022).

The metabolic consequences of obesity in dogs are multifaceted and include a pro-inflammatory state mediated by adipose-derived factors, including hypoadiponectinemia, hyperleptinemia, and insulin resistance (Tropf et al., 2017). These metabolic derangements may affect myocardial function through altered mitochondrial metabolism, localized cardiac insulin resistance, and intramyocardial lipid accumulation (Chess & Stanley, 2008; Lopaschuk et al., 2007; Park et al., 2005). Structural cardiac changes, including myocardial hypertrophy and elevated arterial pressure, have been documented in obese dogs without identifiable primary causes, suggesting that metabolic disturbances may play an independent role in their development (Chandler, 2016; Mehlman et al., 2013).

Despite the growing recognition of obesity as a systemic and metabolically active condition, the extent to which these alterations translate into measurable cardiovascular changes in overweight and obese dogs remains incompletely characterized. Studies evaluating cardiovascular parameters in obese dogs have reported variable findings, with some demonstrating left ventricular hypertrophy, diastolic dysfunction, and altered cardiac sympathovagal balance, while others have found few or no significant echocardiographic differences (Mehlman et al., 2013; Partington et al., 2022; Pongkan et al., 2020; Tropf et al., 2017). These discrepancies highlight the need for studies that integrate clinical, morphometric, electrocardiographic, and echocardiographic parameters to better characterize the early cardiovascular impact of excess body condition in dogs.

Further, most published studies compare only two groups (obese and ideal weight) and do not include an intermediate overweight category, limiting the ability to detect progressive cardiovascular changes across the spectrum of body conditions. Studies conducted in populations representative of canine patients attending primary care practices in Brazil are also scarce; mixed-breed dogs predominate, and the clinical profile may differ from that of populations studied elsewhere.

Therefore, the present study aimed to evaluate and compare the clinical, morphometric, electrocardiographic, and echocardiographic cardiovascular parameters among dogs with normal body condition, overweight, and obesity to identify early markers of cardiovascular involvement associated with increasing adiposity.

## Material and methods

### Study design and ethics

This prospective cohort study included data obtained from patients who attended the Veterinary Hospital of the Federal Rural University of Rio de Janeiro (UFRRJ). The study protocol was approved by the Institutional Animal Care and Use Committee (IACUC) (protocol number 8575191022). Written informed consent was obtained from all owners prior to the inclusion of their animals in the study.

### Animals and eligibility criteria

Dogs aged between 1 and 6 years with body condition scores (BCSs) of 4–9 on the nine-point Laflamme scale were included, regardless of sex or reproductive status. All animals were confirmed to be free of *Dirofilaria immitis* infection based on preventive history and diagnostic testing. Dogs were excluded if they presented with echocardiographically confirmed cardiac disease associated with left atrial enlargement or congestive heart failure, or if there was a clinical suspicion or diagnosis of hypothyroidism or hyperadrenocorticism. These conditions could independently affect cardiac structure and function.

### Clinical evaluation and body condition assessment

All dogs underwent a comprehensive clinical evaluation, including detailed history taking and physical examination, with particular emphasis on cardiorespiratory assessment. The clinical signs evaluated included exercise intolerance, dyspnea, mucous membrane color, capillary refill time, cardiac murmur, cardiac rhythm, lung sounds, and cough reflex.

Body height was measured using a flexible measuring tape from the atlanto-occipital joint to the last sacral vertebra, as described by Muller et al. (2008). The canine body mass index (CBMI) was calculated using an adaptation of the human body mass index equation (Muller et al. 2008):


$$CBMI=\frac{body\;weight\;\left(kg\right)}{height^{2}\;\left(m^{2}\right)}$$ (1)

Thoracic circumference (TC) and abdominal circumference (AC) were measured at the points of greatest diameter using the same flexible tape, and the TC/AC ratio was subsequently calculated. Systolic arterial pressure was measured using the indirect Doppler method with an appropriate cuff size.

### Electrocardiographic evaluation

Electrocardiographic recordings were obtained using InCardio™ Duo software and InCardio™ hardware. The dogs were positioned in right lateral recumbency on a rubber surface, electrodes were attached to the thoracic and pelvic limbs, and 70% alcohol was applied to improve electrical conduction. A paper speed of 25 mm/s and calibration of 1 mV/cm were used. The following parameters were measured in lead II: heart rate (bpm), P-wave duration (ms) and amplitude (mV), PR interval (ms), QRS duration (ms), R-wave amplitude (mV), QT interval (ms), T-wave amplitude (mV), mean electrical axis of P and QRS, and cardiac rhythm.

### Echocardiographic evaluation

Echocardiographic examination was performed using a water-based coupling gel and transducer positioned at standard imaging windows in accordance with the recommendations of the Echocardiography Committee of the American College of Veterinary Internal Medicine, with modifications proposed by Madron (2016). Two-dimensional, M-mode, and Doppler evaluations were performed. Cardiac chambers, wall thickness, and valvular function were assessed. The left atrium (LA) and aortic root (Ao) diameters were measured using a two-dimensional short-axis view, and the left atrium-to-aortic root ratio (LA/Ao) ratio was calculated accordingly. Systolic function was assessed using fractional shortening (FS) and ejection fraction (EF). Diastolic function was evaluated using transmitral Doppler flow: the measurements included E-wave and A-wave peak velocities and the E/A ratio. The maximum aortic and pulmonary outflow velocities and pressure gradients were also obtained. Valvular morphology and the presence of regurgitation were assessed qualitatively using color Doppler.

### Sample size and group allocation

The sample size (n = 30) was calculated using G*Power 3.1 software. Subsequently, a finite population correction was applied using the formula based on previously reported prevalence data (Porsani et al., 2020):


$$N_{adjusted}=\frac{N\;\times n}{N+n}$$ (2)

Animals were categorized into three groups according to the BCS: Group 1 (G1, control) consisting of dogs with normal BCS (4–5/9, n = 10); Group 2 (G2) comprising overweight dogs (BCS 6–7/9, n = 10); and Group 3 (G3) comprising obese dogs (BCS 8–9/9, n = 10).

### Statistical analysis

Statistical analyses were performed using Python (v3.12) with the SciPy library (v1.13). Data distribution was assessed using the Shapiro–Wilk test. Normally distributed continuous variables are presented as mean ± standard deviation and were compared across groups using one-way analysis of variance with Bonferroni post hoc correction. Non-normally distributed variables are presented as median (Q1–Q3) and were compared using the Kruskal–Wallis test, followed by pairwise Mann–Whitney U tests with Bonferroni correction. For multiple pairwise comparisons (three groups), the adjusted significance threshold was set at p < 0.017. Categorical variables were compared using the chi-squared test of independence or Fisher's exact test when expected cell frequencies were below 5. Statistical significance was set at p < 0.05 for all analyses.

## Results

Thirty dogs were enrolled and equally distributed into three groups of ten animals each. Group 1 (G1, normal body condition, BCS 4–5/9) comprised six females (60%) and four males (40%) with a median age of 5.0 years (Q1–Q3: 4.0–6.0) and a median body weight of 5.0 kg (3.0–8.0). Seven animals were sexually intact (70%), and three were neutered (30%). The breeds represented in G1 included mixed-breed dogs (n = 3), Pinschers (n = 2), Yorkshire Terriers (n = 2), Poodles (n = 1), Miniature Poodles (n = 1), and Lhasa Apsos (n = 1).

Group 2 (G2, overweight, BCS 6–7/9) comprised seven females (70%) and three males (30%) with a median age of 5.5 years (5.0–6.0) and a median body weight of 19.5 kg (9.25–28.0). Four animals were intact (40%), and six were neutered (60%). Most dogs in this group were mixed-breed (n = 8), with one Shih Tzu and one Yorkshire Terrier.

Group 3 (G3, obese, BCS 8–9/9) comprised seven females (70%) and three males (30%) with a median age of 6.0 (5.0–6.0) years and a median body weight of 25.0 (21.0–31.0) kg. Five animals were intact (50%), and five were neutered (50%). The breeds included mixed-breed dogs (n = 5), Pit Bull Terriers (n = 2), Labrador Retrievers (n = 1), Chihuahuas (n = 1), and Poodles (n = 1). Mixed-breed dogs were predominant across the entire sample (n = 16/30, 53%).

No statistically significant differences were observed between the groups in sex distribution (p = 0.86), age (p = 0.54), or reproductive status (p = 0.39), indicating homogeneity of these baseline characteristics across groups.

The CBMI increased progressively across groups -G1: 10.80 (9.54–13.79); G2: 17.72 (15.38–19.70); and G3: 20.66 (18.14–21.76). It also differed significantly between G1 and G2 (p = 0.012) and between G1 and G3 (p < 0.0001), but not between G2 and G3 (p = 0.30; Table 1). The TC/AC ratio was significantly lower for overweight and obese dogs than for normal animals (p = 0.003; G1 vs G2: p = 0.019; G1 vs G3: p = 0.005), with no significant difference between G2 and G3 (p = 1.00). This reflected progressive abdominal fat redistribution with increasing adiposity.

**Table 1:** Descriptive statistics and comparison of continuous cardiovascular parameters among dogs classified as normal (G1), overweight (G2), and obese (G3).

**Variable**	**Normal (G1)**	**Overweight (G2)**	**Obese (G3)**	**Test**	**p-value**	**G1 vs G2**	**G1 vs G3**	**G2 vs G3**
**Clinical and anthropometric parameters**								
Age (years)	5.00 (4.00–6.00)	5.50 (5.00–6.00)	6.00 (5.00–6.00)	KW	0.5406	—	—	—
Body weight (kg)	5.00 (3.00–8.00)	19.50 (9.25–28.00)	25.00 (21.00–31.00)	KW	0.0004^*^	0.0156*	0.0004*	0.8931
BCS (×/9)	5.00 (4.00–5.00)	7.00 (6.25–7.00)	8.00 (8.00–8.00)	KW	<0.0001*	0.0333*	<0.0001*	0.0333*
CBMI	10.80 (9.54–13.79)	17.72 (15.38–19.70)	20.66 (18.14–21.76)	KW	<0.0001*	0.0123*	<0.0001*	0.2962
Heart rate – clinical (bpm)	121.60 ± 18.59	123.40± 20.31	114.20 ± 14.62	AnV	0.4894	—	—	—
CRT (s)	2.00 (2.00–2.00)	2.00 (2.00–2.50)	2.00 (2.00–2.00)	KW	0.8920	—	—	—
SAP (mmHg)	165.00 (150.00–189.00)	150.00 (130.00–160.00)	155.00 (140.00–167.50)	KW	0.1162	—	—	—
TC/AC ratio	1.31 (1.25–1.42)	1.17 (1.11–1.19)	1.13 (1.06–1.15)	KW	0.0027*	0.0190*	0.0045*	1.0000
**Electrocardiographic parameters**								
Mean HR – ECG (bpm)	119.40 ± 33.12	109.90 ± 23.77	110.00 ± 21.22	AnV	0.6867	—	—	—
P-wave duration (ms)	34.00 (32.50–39.00)	40.00 (38.00–40.00)	40.00 (40.00–40.00)	KW	0.0297*	0.1427	0.0604	1.0000
QRS duration (ms)	52.00 (50.50–55.50)	56.00 (54.00–64.00)	59.00 (56.00–63.00)	KW	0.0490*	0.1857	0.0668	1.0000
QT interval (ms)	192.50 ± 28.25	200.90 ± 12.37	195.80 ± 19.88	AnV	0.6748	—	—	—
P-wave amplitude (mV)	0.25 ± 0.10	0.22 ± 0.07	0.21 ± 0.06	AnV	0.8163	—	—	—
R-wave amplitude (mV)	1.20 (0.95–1.87)	1.39 (0.96–1.54)	1.50 (1.26–1.79)	KW	0.3714	—	—	—
T-wave amplitude (mV)	0.05 ± 0.24	-0.05 ± 0.32	0.01 ± 0.18	AnV	0.3618	—	—	—
P-wave axis (°)	55.75 (50.76–67.85)	69.29 (62.80–78.70)	62.75 (57.34–73.79)	KW	0.0548	—	—	—
QRS axis (°)	61.96 (51.56–73.84)	71.44 (41.53–77.47)	73.30 (65.37–80.84)	KW	0.7166	—	—	—
**Echocardiographic parameters**								
LA diameter (cm)	1.81 ± 0.47	1.81 ± 0.31	2.47 ± 0.67	AnV	0.0095*	1.0000	0.0641	0.0360*
Aortic root diameter (cm)	1.40 ± 0.23	1.49 ± 0.22	1.62 ± 0.44	AnV	0.1301	—	—	—
LA/Ao ratio	1.23 (1.15–1.32)	1.33 (1.28–1.38)	1.40 (1.37–1.42)	KW	0.0108*	1.0000	0.0105*	0.1153
Aortic Vmáx (cm/s)	122.71 ± 30.74	135.34 ± 19.71	121.97 ± 20.37	AnV	0.3927	—	—	—
Aortic ΔP (mmHg)	5.72 (5.19–7.29)	6.02 (5.47–9.35)	5.41 (5.18–5.83)	KW	0.3511	—	—	—
Pulmonary Vmáx (cm/s)	99.88 ± 18.94	96.74 ± 16.08	104.22 ± 22.73	AnV	0.6925	—	—	—
Pulmonary ΔP (mmHg)	3.26 (2.83–3.91)	3.63 (3.17–5.42)	4.83 (3.54–5.34)	KW	0.2732	—	—	—
Transmitral E wave (cm/s)	69.06 (61.98–73.93)	75.77 (73.25–78.01)	77.78 (71.07–94.48)	KW	0.0912	—	—	—
Transmitral A wave (cm/s)	48.07 ± 28.03	57.92 ± 12.41	66.47 ± 19.59	AnV	0.1658	—	—	—
E/A ratio	1.37 ± 0.40	1.34 ± 0.31	1.39 ± 0.44	AnV	0.9692	—	—	—

* p < 0.05. A, late diastolic wave; ANOVA, one-way analysis of variance; Ao, aortic root; BCS, body condition score; CBMI, canine body mass index; CRT, capillary refill time; E, early diastolic wave; E/A, E-to-A velocity ratio; HR, heart rate; KW, Kruskal-Wallis test; LA, left atrium; LA/Ao, left atrium-to-aortic root ratio; SAP, systolic arterial pressure; TC/AC, thoracic-to-abdominal circumference ratio; Vmáx, maximum velocity; ΔP, pressure gradient.

Data are expressed as mean ± SD (ANOVA) or median (Q1–Q3) (KW). Post-hoc p-values (Bonferroni correction) are shown only for significant variables. Highlighted rows indicate statistically significant results (p < 0.05).

No significant differences were observed between the groups for heart rate (p = 0.49), capillary refill time (p = 0.89), or systolic arterial pressure (p = 0.12). Full descriptive data are presented in Table 1.

Electrocardiographic evaluation revealed significant differences in P-wave duration (p = 0.030) and QRS complex duration (p = 0.049) across groups (Table 1). The median P-wave duration increased progressively from G1 (34.00 ms [32.50–39.00]) through G2 (40.00 ms [38.00–40.00]) to G3 (40.00 ms [40.00–40.00]), and the QRS duration followed a similar trend: G1 (52.00 ms [50.50–55.50]), G2 (56.00 ms [54.00–64.00]) and G3 (59.00 ms [56.00–63.00]). However, post hoc pairwise comparisons did not identify a statistically significant difference in any pair after Bonferroni correction (all pairwise p > 0.017). The QT interval, wave amplitudes (P, R, and T), mean heart rate on ECG, P-wave axis, and QRS axis did not differ significantly between the groups (all p > 0.05).

Echocardiographic analysis demonstrated a significant increase in the left atrial (LA) diameter in obese dogs relative to overweight animals (G2 vs. G3: p = 0.036; Table 1). The mean LA diameter was 1.81 ± 0.47 cm for G1, 1.81 ± 0.31 cm for G2, and 2.47 ± 0.67 cm for G3. The LA/Ao ratio also differed significantly across groups (p = 0.011), and a post-hoc analysis confirmed a significant difference between G1 and G3 (p = 0.011). No significant difference was found between G1 and G2 (p = 1.00) or between G2 and G3 (p = 0.12). The median LA/Ao ratio was 1.23 (1.15–1.32) for G1, 1.33 (1.28–1.38) for G2, and 1.40 (1.37–1.42) in G3. The aortic root diameter, Doppler flow velocities and pressure gradients for both the aortic and pulmonary outflow tracts, and transmitral flow indices, including E-wave, A-wave, and E/A ratio, did not differ significantly across groups (all p > 0.05).

Exercise intolerance was reported in 2/10 (20%), 6/10 (60%), and 9/10 (90%) of dogs in groups G1, G2, and G3, respectively (p = 0.007). Dyspnea was significantly more frequent in obese dogs (G3:7/10, 70%) than in normal (G1:1/10, 10%) and overweight animals (G2:1/10, 10%; p = 0.003). Mitral valve abnormalities differed significantly among the groups (p = 0.023), with mitral valve enlargement observed in 1/10 (10%), 1/10 (10%), and 4/10 (40%) of dogs in groups G1, G2, and G3, respectively. Mitral insufficiency was more prevalent in group G2 (5/10, 50%). No significant associations were found between groups for cardiac murmur, cardiac rhythm, lung sounds, or the remaining echocardiographic qualitative variables (all p > 0.05). Categorical data are presented in Table 2.

**Table 2:** Frequency distribution and categorical clinical and echocardiographic variables among dogs classified as normal (G1), overweight (G2), and obese (G3).

**Variable / Category**	**Normal (G1)**	**Overweight (G2)**	**Obese (G3)**	**p-value**	**Test**
Sex				0.8607	χ^2^ / Fisher
Female	6 (60%)	7 (70%)	7 (70%)		
Male	4 (40%)	3 (30%)	3 (30%)		
Reproductive status				0.3916	χ^2^ / Fisher
Intact	7 (70%)	4 (40%)	5 (50%)		
Neutered	3 (30%)	6 (60%)	5 (50%)		
Heartworm prevention				0.7866	χ^2^ / Fisher
Yes	2 (20%)	2 (20%)	1 (10%)		
No	8 (80%)	8 (80%)	9 (90%)		
Exercise intolerance				0.0066*	χ^2^ / Fisher
Yes	2 (20%)	6 (60%)	9 (90%)		
No	8 (80%)	4 (40%)	1 (10%)		
Dyspnea				0.0033*	χ^2^ / Fisher
Yes	1 (10%)	1 (10%)	7 (70%)		
No	9 (90%)	9 (90%)	3 (30%)		
Cardiac murmur				0.3661	χ^2^ / Fisher
Present	5 (50%)	2 (20%)	4 (40%)		
Absent	5 (50%)	8 (80%)	6 (60%)		
Cardiac rhythm				0.8747	χ^2^ / Fisher
Normal	5 (50%)	6 (60%)	5 (50%)		
Abnormal	5 (50%)	4 (40%)	5 (50%)		
Lung sounds				0.0907	χ^2^ / Fisher
Normal	4 (40%)	8 (80%)	8 (80%)		
Abnormal	6 (60%)	2 (20%)	2 (20%)		
Mitral valve (echo)				0.0231^*^	χ^2^ / Fisher
Normal	8 (80%)	4 (40%)	6 (60%)		
Insufficient	1 (10%)	5 (50%)	0 (0%)		
Enlarged	1 (10%)	1 (10%)	4 (40%)		
Tricuspid valve (echo)				0.0965	χ^2^ / Fisher
Normal	9 (90%)	3 (30%)	5 (50%)		
Insufficient	0 (0%)	1 (10%)	0 (0%)		
Aortic valve (echo)				1.0000	χ^2^ / Fisher
Normal	10 (100%)	10 (100%)	10 (100%)		
Pulmonary valve (echo)				0.3554	χ^2^ / Fisher
Normal	10 (100%)	10 (100%)	9 (90%)		

Data expressed as n (%). χ^2^, chi-squared test; Fisher, Fisher's exact test applied according to expected cell counts < 5). Highlighted rows indicate statistically significant results.

* p < 0.05.

## Discussion

The present study evaluated clinical, electrocardiographic, and echocardiographic parameters in dogs with normal body condition, overweight, and obesity, aiming to characterize the cardiovascular profile across body condition categories. The main findings were as follows: (i) exercise intolerance and dyspnea were significantly more frequent in obese dogs; (ii) the CBMI and TC/AC ratio differed significantly across groups, reflecting progressive morphometric changes associated with adiposity; (iii) electrocardiographic changes, particularly prolonged P-wave and QRS duration, were observed; (iv) left atrial diameter and LA/Ao ratio were significantly greater in obese dogs than in normal animals; and (v) mitral valve abnormalities were more prevalent in G3. Together, these findings suggest that obesity in dogs is associated with early cardiovascular alterations, even in the absence of overt cardiac dysfunction.

Exercise intolerance was reported in 20%, 60%, and 90% of dogs in G1, G2, and G3, respectively (p = 0.007), and dyspnea was significantly more frequent in obese dogs (G3:70%) than in normal and overweight animals (10% each; p = 0.003). These findings are consistent with the well-documented impact of excess adiposity on cardiorespiratory performance in dogs. Increased fat deposition reduces thoracic compliance, elevates diaphragmatic pressure, and increases the work of breathing, all of which contribute to reduced exercise capacity and the perception of respiratory distress (Bach et al., 2007; Chandler, 2016; Manens et al., 2014). The progressive gradient observed across groups reinforces the dose-dependent nature of these functional impairments and supports the use of exercise intolerance as a practical clinical marker of obesity-related cardiorespiratory burden in dogs.

The TC/AC ratio was significantly lower in G2 and G3 than in G1 (p = 0.003; G1:1.31, G2:1.17, G3:1.13), with no significant difference between the overweight and obese groups. In dogs with an ideal body condition, the thoracic circumference normally exceeds the abdominal circumference, producing a characteristic hourglass silhouette. With increasing adiposity, fat accumulates preferentially in the abdominal, sublumbar, and inguinal regions, causing the abdominal circumference to increase disproportionately relative to the thoracic circumference and progressively reducing the TC/AC ratio toward unity. Morphometric studies involving dogs have confirmed that abdominal circumference changes more markedly than thoracic circumference with weight gain, and the ratio between these measurements correlates strongly with the BCS and estimated body fat percentage (Witzel et al., 2014). Chun et al. (2019) reported that an abdominal-to-thoracic girth ratio of approximately 1.0 is associated with an ideal body condition, with values above 1.05 indicating overweight or obesity. This revealed the inverse pattern observed in the present study. The absence of a significant difference between G2 and G3 suggests that this morphometric ratio may plateau at moderate obesity, possibly reflecting concurrent enlargement of both thoracic and abdominal fat depots. Together with the CBMI (p < 0.0001), the TC/AC ratio provides a simple, non-invasive morphometric complement to BCS for quantifying adiposity distribution in clinical practice.

Electrocardiographic assessment revealed significant differences in P-wave (p = 0.030) and QRS complex (p = 0.049) durations across groups, with both parameters showing a progressive increase from G1 through G3. The pairwise post-hoc comparisons did not reach individual significance after Bonferroni correction, likely reflecting the limited statistical power of a sample of ten animals per group, but the overall trend is clinically meaningful. Prolonged P-wave duration is a recognized marker of atrial conduction delay and may reflect early left atrial enlargement or interstitial changes secondary to volume overload, which is consistent with the increased LA diameter observed echocardiographically in obese dogs. This relationship has been documented in dogs with myxomatous mitral valve disease, in which P-wave duration and amplitude are significantly correlated with the LA/Ao ratio (Noszczyk-Nowak et al., 2017).

In a large cross-sectional study of more than 12,000 ECGs from apparently healthy dogs, Murphy et al. (2022) confirmed that both P-wave and QRS durations are positively associated with increasing body weight, suggesting that some of the ECG changes observed in the present study may be attributable to body size per se rather than to obesity-specific cardiac remodelling. Pereira-Neto et al. (2010) similarly reported that P-wave duration exceeded reference values in a significant proportion of obese dogs and normalized following weight reduction, suggesting that these conduction changes are at least partially reversible. In the present study, heart rate, QT interval, wave amplitudes, and cardiac axes did not differ significantly between groups, suggesting that the earliest electrocardiographic manifestations of obesity in dogs are subtle and predominantly affect conduction timing rather than impulse amplitude or rhythm.

The most relevant echocardiographic finding was the progressive increase in left atrial diameter and LA/Ao ratio across groups, with a statistically significant difference observed between G1 and G3 for both parameters (p = 0.010 and p = 0.011, respectively). Left atrial enlargement in obese individuals has been attributed to chronic volume overload resulting from expanded plasma volume, increased cardiac output, and elevated preload associated with excess adipose tissue (Tropf et al., 2017). In a study of small-breed dogs, the LA/Ao ratio for obese animals (median 1.39) was numerically higher than that for controls with ideal weights (1.32). The difference did not reach statistical significance, but the authors suggested that LA dilatation may be an early, subclinical manifestation preceding overt diastolic dysfunction. The progressive LA enlargement observed in the present study supports the concept that left atrial remodelling begins during the overweight stage and becomes more pronounced with frank obesity, warranting echocardiographic monitoring even before dogs attain BCSs indicating obesity.

Unlike some previous reports, the present study did not identify significant differences in systolic function indices (EF, FS) or conventional diastolic filling parameters (E-wave, A-wave, E/A ratio) between groups. Partington et al. (2022), in a prospective study of 24 client-owned obese dogs, identified impaired relaxation on tissue Doppler imaging in all dogs and increased left ventricular wall thickness before weight reduction. That study observed a median LA/Ao of 1.28, closely matching the LA/Ao values of 1.33 and 1.40 found in G2 and G3 of the present study, respectively. Tropf et al. (2017) reported significantly higher EF and FS percentages for obese small-breed dogs than for controls with ideal weights. This was interpreted as a hyperdynamic response to increased preload and sympathetic activation. Pongkan et al. (2020) reported the opposite pattern: reduced %EF and %FS in intact male obese dogs, alongside eccentric left ventricular hypertrophy, attributed to increased oxidative stress and cardiac sympathovagal imbalance. These apparently contradictory findings likely reflect differences in the stage of obesity-related cardiac disease, sex and neuter status, breed composition, and the presence or absence of concomitant metabolic derangements. In the present study, the absence of significant systolic or diastolic dysfunction in dogs that nevertheless presented clinical signs and left atrial enlargement suggests that these animals may have had an early, compensated stage of obesity-related cardiac involvement, which is consistent with the findings of Partington et al. (2022) and the natural history of obesity cardiomyopathy in human medicine.

Mitral valve abnormalities differed significantly across groups (p = 0.023), with enlargement of the mitral valve apparatus more prevalent in G3 (40%) than in G1 and G2 (10% each) and mitral insufficiency more frequent in G2 (50%). The echocardiographic assessment of the mitral valve in this study was qualitative, and the presence of mild mitral insufficiency in the overweight group without associated chamber dilatation may represent physiological or incipient regurgitation rather than hemodynamically significant disease. Nonetheless, this association deserves attention, particularly because small-breed dogs, which predominated in G1 and G2 in the present study, are inherently predisposed to myxomatous mitral valve disease, which accounts for more than 70% of all acquired cardiac diseases in dogs and predominantly affects animals weighing less than 20 kg (Keene et al., 2019). Tropf et al. (2017) emphasized that obesity-related cardiac dysfunction may have additive effects in dogs with concurrent degenerative valvular disease, potentially accelerating progression to congestive heart failure. Differentiating between adiposity-related remodelling and early myxomatous mitral valve disease requires a Doppler-based quantitative assessment of regurgitation severity and longitudinal follow-up.

Heart rate did not differ significantly between groups in the present study, but the literature suggests that obesity in dogs may affect cardiac autonomic modulation independently of resting heart rate. Santos Filho et al. (2019), using heart rate variability (HRV) analysis in Beagle dogs, found that obese animals had higher rMSSD (Root Mean Square of Successive Differences) values than the controls with normal weight. This suggest increased parasympathetic tone, a finding that contrasts with the sympathetic predominance described in obese humans. Conversely, Pongkan et al. (2020) demonstrated cardiac sympathovagal imbalance in obese male dogs via an increased LF/HF ratio, and Vieira et al. (2022) reported reduced HRV in mildly overweight dogs specifically using frequency-domain analysis. Partington et al. (2022) found no evidence of decreased HRV in obese dogs, attributing these discrepancies to differences in breed composition and HRV assessment methods. These conflicting results collectively highlight the complexity of autonomic regulation in obese dogs and underscore the need for HRV analysisusing both time and frequency domains as a complementary diagnostic tool. HRV analysis was not performed in the present study.

The limitations of the present study should be acknowledged. The relatively small sample size (n = 10 per group) reduced statistical power, particularly for post-hoc comparisons, and may have prevented the detection of subtle inter-group differences. All included dogs were younger than 7 years, which may have limited the chronicity of cardiovascular exposure necessary for overt structural cardiac changes to develop. The predominance of mixed-breed and small-breed dogs, while representative of the clinical population attending primary care practices, introduces breed-related variability that was not controlled for. The echocardiographic evaluation relied on conventional M-mode and two-dimensional parameters; more sensitive techniques such as myocardial strain analysis by speckle-tracking echocardiography were not performed and could potentially identify subclinical myocardial dysfunction not captured by standard indices (Pongkan et al., 2020). The qualitative assessment of valvular function precludes quantitative grading of regurgitation severity. Further, metabolic parameters, including insulin resistance markers, lipid profiles, and adipokine concentrations, were not evaluated, limiting the ability to link cardiovascular findings to underlying metabolic derangements (Tropf et al., 2017). Longitudinal studies with larger, breed-controlled cohorts and comprehensive metabolic profiling are needed to further elucidate the temporal relationship between increasing adiposity and the development of structural and functional cardiovascular disease in dogs.

## Conclusion

The findings of the present study indicate that overweight and obesity in dogs are associated with early cardiovascular manifestations, including exercise intolerance, dyspnea, changes in electrocardiographic conduction parameters, progressive left atrial enlargement, reduction in the TC/AC ratio, and increased prevalence of mitral valve abnormalities. These alterations were present in the absence of overt cardiac dysfunction, suggesting a compensated stage of obesity-related cardiovascular involvement. Early clinical recognition of these signs, combined with echocardiographic monitoring and body condition assessment, may support the implementation of preventive strategies, particularly weight management, before irreversible cardiac remodelling occurs. Future studies should incorporate metabolic profiling, HRV analysis, and myocardial strain imaging to more comprehensively characterize the cardiovascular consequences of obesity across different body condition stages in dogs.

## References

[B001] Bach J. F., Rozanski E. A., Bedenice D., Chan D. L., Freeman L. M., Lofgren J. L., Oura T. J., Hoffman A. M. (2007). Association of expiratory airway dysfunction with marked obesity in healthy adult dogs. American Journal of Veterinary Research.

[B002] Chandler M. L. (2016). Impact of obesity on cardiopulmonary disease. The Veterinary Clinics of North America. Small Animal Practice.

[B003] Chess D. J., Stanley W. C. (2008). Role of diet and fuel overabundance in the development and progression of heart failure. Cardiovascular Research.

[B004] Chun J. L., Kim S., Ji S. Y., Nam A., Kim B., Lee B., Kim B. H. (2019). A simple method to evaluate body condition score to maintain the optimal body weight in dogs. Journal of Animal Science and Technology.

[B005] Courcier E. A., Thomson R. M., Mellor D. J., Yam P. S. (2010). An epidemiological study of environmental factors associated with canine obesity. The Journal of Small Animal Practice.

[B006] Endenburg N., Soontararak S., Charoensuk C., van Lith H. A. (2018). Quality of life and owner attitude to dog overweight and obesity in Thailand and the Netherlands. BMC Veterinary Research.

[B007] German A. J. (2006). The growing problem of obesity in dogs and cats. The Journal of Nutrition.

[B008] Keene B. W., Atkins C. E., Bonagura J. D., Fox P. R., Häggström J., Fuentes V. L., Oyama M. A., Rush J. E., Stepien R., Uechi M. (2019). ACVIM consensus guidelines for the diagnosis and treatment of myxomatous mitral valve disease in dogs. Journal of Veterinary Internal Medicine.

[B009] Lopaschuk G. D., Folmes C. D. L., Stanley W. C. (2007). Cardiac energy metabolism in obesity. Circulation Research.

[B010] Madron E., Chetboul V., Bussadori C., Madron E. (2016). Clinical echocardiography of the dog and cat.

[B011] Manens J., Ricci R., Damoiseaux C., Gault S., Contiero B., Diez M., Clercx C. (2014). Effect of body weight loss on cardiopulmonary function assessed by 6-minute walk test and arterial blood gas analysis in obese dogs. Journal of Veterinary Internal Medicine.

[B012] Mao J., Xia Z., Chen J., Yu J. (2013). Prevalence and risk factors for canine obesity surveyed in veterinary practices in Beijing, China. Preventive Veterinary Medicine.

[B013] Mehlman E., Bright J. M., Jeckel K., Porsche C., Veeramachaneni D. N. R., Frye M. (2013). Echocardiographic evidence of left ventricular hypertrophy in obese dogs. Journal of Veterinary Internal Medicine.

[B014] Montoya-Alonso J. A., Bautista-Castaño I., Peña C., Suárez L., Juste M. C., Tvarijonaviciute A. (2017). Prevalence of canine obesity, obesity-related metabolic dysfunction, and relationship with owner obesity in an obesogenic region of Spain. Frontiers in Veterinary Science.

[B015] Muller D. C. D. M., Schossler J. E., Pinheiro M. (2008). Adaptação do índice de massa corporal humano para cães. Ciência Rural.

[B016] Murphy L., Nakamura R., Gentile-Solomon J., Spake A., Szlosek D. (2022). Assessment of age, gender, and anxiety on ECG waveform morphology in a large population of domestic dogs. Scientific Reports.

[B017] Noszczyk-Nowak A., Szałas A., Pasławska U., Nicpoń J. (2017). Diagnostic accuracy of electrocardiographic P wave related parameters in the assessment of left atrial size in dogs with degenerative mitral valve disease. BMC Veterinary Research.

[B018] Park S. Y., Cho Y. R., Kim H. J., Higashimori T., Danton C., Lee M. K., Dey A., Bultman S., Bhaskara S., Bhaskara S., Kim J. K. (2005). Unraveling the temporal pattern of diet-induced insulin resistance in individual organs and cardiac dysfunction in C57BL/6 mice. Diabetes.

[B019] Partington C., Hodgkiss-Geere H., Woods G. R. T., Dukes-McEwan J., Flanagan J., Biourge V., German A. J. (2022). The effect of obesity and subsequent weight reduction on cardiac structure and function in dogs. BMC Veterinary Research.

[B020] Pereira-Neto G. B., Brunetto M. A., Sousa M. G., Carciofi A. C., Camacho A. A. (2010). Effects of weight loss on the cardiac parameters of obese dogs. Pesquisa Veterinária Brasileira.

[B021] Pongkan W., Jitnapakarn W., Phetnoi W., Punyapornwithaya V., Boonyapakorn C. (2020). Obesity-induced heart rate variability impairment and decreased systolic function in obese male dogs. Animals.

[B022] Porsani M. Y. H., Teixeira F. A., Oliveira L. A. C. C., Pedrinelli V., Dias R. A., German A. J., Brunetto M. A. (2020). Prevalence of canine obesity in the city of São Paulo, Brazil. Scientific Reports.

[B023] Santos M., Hainfellner D. C., Lemos N. M. O., Mendes C. O. F., Malandrim P., Campos J. M., Ballot S., Oliveira P. C., Paiva J. P. (2019). Influence of weight loss on heart rate variability in dogs. Brazilian Journal of Veterinary Medicine.

[B024] Thomson R. L., Lester N. V., Cole G. L., Webb J. A., Hicks B. (2022). Canine obesity - prevalence, risk factors and impact: A narrative review. Veterinary Evidence.

[B025] Tropf M., Nelson O. L., Lee P. M., Weng H. Y. (2017). Cardiac and metabolic variables in obese dogs. Journal of Veterinary Internal Medicine.

[B026] Vieira A. B., Restrepo M. A., Auzenne D., Molina K., O’Sullivan M., Machado M. V., Cavanaugh S. M. (2022). Mild to moderate overweight in dogs: Is there an impact on routine hematological and biochemical profiles, echocardiographic parameters and cardiac autonomic modulation?. Veterinary Research Communications.

[B027] Witzel A. L., Kirk C. A., Henry G. A., Toll P. W., Brejda J. J., Paetau-Robinson I. (2014). Use of a novel morphometric method and body fat index system for estimation of body composition in overweight and obese dogs. Journal of the American Veterinary Medical Association.

[B028] Zoran D. L. (2010). Obesity in dogs and cats: A metabolic and endocrine disorder. The Veterinary Clinics of North America. Small Animal Practice.

